# Head capsule characters in the Hymenoptera and their phylogenetic implications

**DOI:** 10.3897/zookeys.130.1438

**Published:** 2011-09-24

**Authors:** Lars Vilhelmsen

**Affiliations:** 1 Natural History Museum of Denmark, University of Copenhagen, Universitetsparken 15, DK-2100, Denmark

**Keywords:** Hymenoptera, morphology, phylogeny, head anatomy

## Abstract

The head capsule of a taxon sample of three outgroup and 86 ingroup taxa is examined for characters of possible phylogenetic significance within Hymenoptera. 21 morphological characters are illustrated and scored, and their character evolution explored by mapping them onto a phylogeny recently produced from a large morphological data set. Many of the characters are informative and display unambiguous changes. Most of the character support demonstrated is supportive at the superfamily or family level. In contrast, only few characters corroborate deeper nodes in the phylogeny of Hymenoptera.

## Introduction

Phylogenetic research on Hymenoptera has been pursued for more than a century (see Sharkey 2007 for an overview). However, only for the past forty-odd years have this effort been more focused by the application of cladistic methodology and active search for characters that might be useful for elucidating hymenopteran evolution. (Rasnitsyn (1969, 1980, 1988) made ground breaking advances in the field by surveying characters across the entire Hymenoptera, extant as well as extinct. His culminating effort was a widely cited phylogenetic hypothesis for the order (Rasnitsyn 1988). Although the analyses were not performed in a strictly cladistic context, the phylogenetic treatment presented in this paper formed a landmark. It served as an inspiration as well as a characters source for the first more stringent analyses of hymenopteran relationships (Ronquist et al. 1999; Carpenter and Wheeler 1999; Dowton and Austin 2001). Not all the relationships proposed by Rasnitsyn (1988) have been corroborated by these and later efforts, and the phylogenetics of Hymenoptera continuously evolve as morphological character systems are re-evaluated and expanded (e.g., Vilhelmsen et al. 2010a) and molecular data sets are developed (e.g., Castro and Dowton 2006; Sharanowski et al. 2010; Heraty et al. 2011). However, the hypotheses presented by Rasnitsyn continue to be relevant when comparing and discussing the results of more recent analyses.

Characters from the head capsule have long been included in attempts to make phylogenetic inferences across the Hymenoptera. Ross (1937) discussed some of the variation in this region among basal Hymenoptera, in particular the different configurations of ventral sclerotisations occurring between the occipital and oral foramina. However, as he was not working in a cladistic context, he was unable to establish putative transformation series and character support for different clades. In contrast, Rasnitsyn (1988) explicitly employed head capsule characters, among others. This was continued in the first stringently cladistic analyses performed for the basal hymenopteran lineages by (Vilhelmsen (1997, 2001) and Schulmeister (2003a, b). These analyses included additional characters discussed in (Vilhelmsen (1996, 1997, 1999). In the latter paper, the variation within the ventral sclerotisations among especially basal Hymenoptera was discussed at length and the difficulties with establishing homologies and inferring transitions between different conditions in this particular feature was highlighted.

In the present paper, I review characters from the head capsule from previous treatments as well as introducing some new ones. The characters are scored from a taxon sample closely matching that of Vilhelmsen et al. (2010a), which used a comprehensive data set from the skeleto-musculature of the mesosoma to analyse phylogenetic relationships broadly across the Hymenoptera. This data set was assembled as part of the US National Science Foundation Hymenoptera Assembling the Tree of Life project. Indeed, the characters presented here will be included in forthcoming combined analyses of molecular and morphological data. In the present paper, the characters scored will be mapped on a phylogeny from Vilhelmsen et al. (2010a) and their possible phylogenetic implications discussed.

## Materials and methods

**Figure 1.**
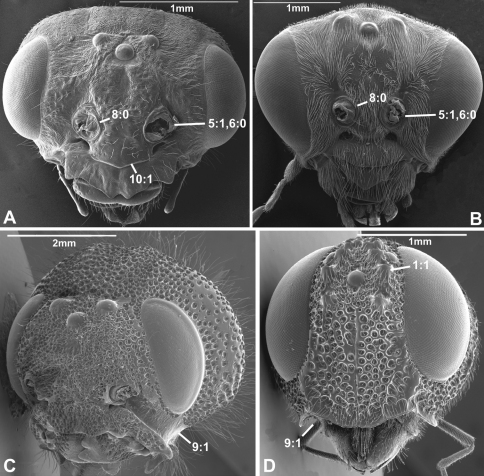
Anterior view of head capsule of **A** *Macroxyela ferruginea* (Xyeloidea, Xyelidae); modified from MB 102882 **B** *Athalia rosae* (Tenthredinoidea, Tenthredinidae); modified from MB 102486 **C** *Tremex columba* (Siricoidea, Siricidae); modified from MB 134704 **D** *Orussus abietinus* (Orussoidea, Orussidae); modified from MB 134672. Numbers indicate character numbers:character states.

**Figure 2.**
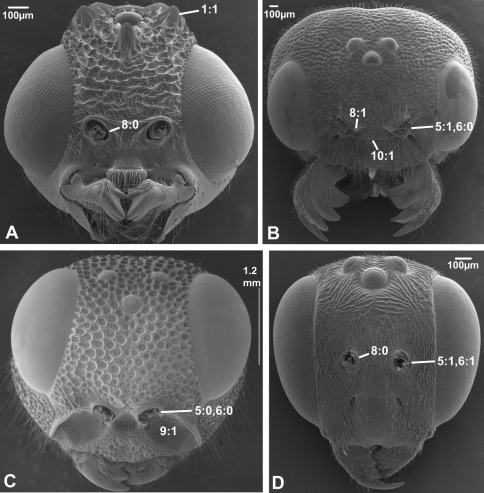
Anterior view of head capsule of **A** *Megischus* sp. (Stephanoidea, Stephanidae), modified from MB 103607 **B** *Taeniogonalos gundlachii* (Trigonaloidea, Trigonalidae); modified from MB 103032 **C** *Megalyra fasciipennis* (Megalyroidea, Megalyridae); modified from MB 103568 **D** *Pseudofoenus* sp. (Evanioidea, Gasteruptiidae); modified from MB 103788. Numbers indicate character numbers:character states.

**Figure 3.**
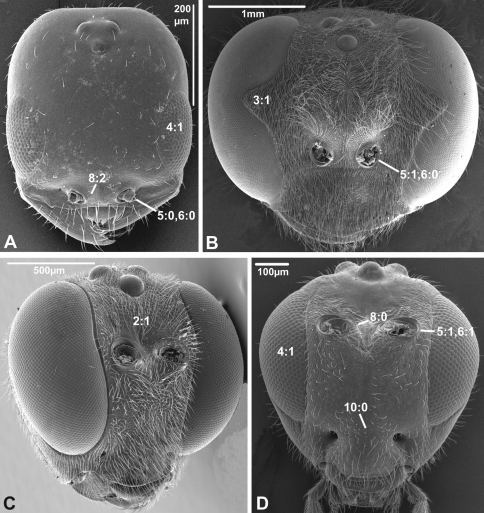
Anterior view of head capsule of **A** *Cephalonomia stephanoderis* (Chrysidoidea, Bethylidae); modified from MB 102531 **B** *Pison chilense* (Apoidea, Crabronidae); modified from MB 102586 **C** *Dusona egregia* (Ichneumonoidea, Ichneumonidae); modified from MB 134625 **D** *Orgilus gracilis* (Ichneumonoidea, Braconidae); modified from MB 103649. Numbers indicate character numbers:character states.

**Figure 4.**
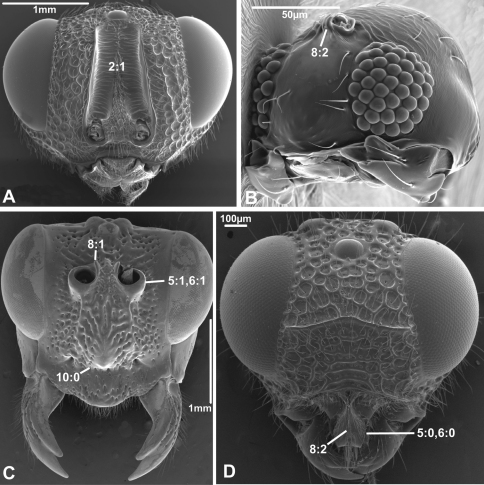
Anterior view of head capsule of **A** *Acanthochalcis* sp. (Chalcidoidea, Chalcididae), modified from MB 134808 **B** *Mymaromma anomalum* (Mymarommatoidea, Mymarommatidae); modified from MB 101797 **C** *Pelecinus polyturator* (Pelecinidae); modified from MB 79061 **D** *Sparasion formosum* (Platygastridae); modified from MB 103049. Numbers indicate character numbers:character states.

**Figure 5.**
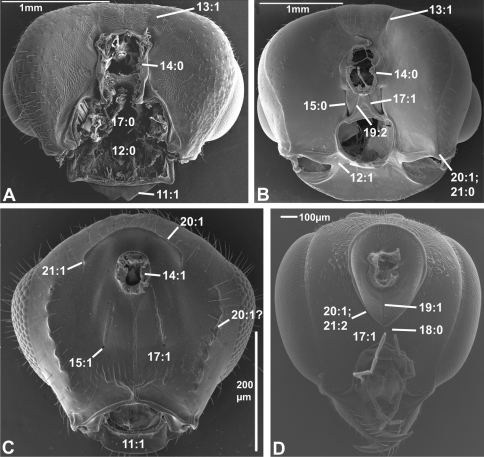
Posterior view of head capsule of **A** *Macroxyela ferruginea* (Xyeloidea, Xyelidae)¸ modified from MB 102885 **B** *Onycholyda amplecta* (Pamphilioidea, Pamphiliidae); modified from MB 102837 **C** *Ceraphron* sp. (Ceraphronoidea, Ceraphronidae); modified from MB 78643 **D** *Pseudofoenus* sp. (Evanioidea, Gasteruptiidae); modified from MB 103810. Numbers indicate character numbers:character states.

**Figure 6.**
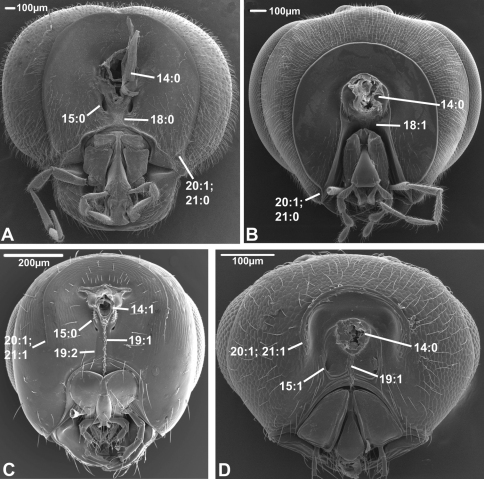
Posterior view of head capsule of **A** *Taeniogonalos gundlachii* (Trigonaloidea, Trigonalidae); modified from MB 103043 **B** *Dusona egregia* (Ichneumonoidea, Ichneumonidae); modified from MB 103363 **C** *Megastigmus transvaalensis* (Chalcidoidea, Torymidae); modified from MB 101672 **D** *Isostasius* sp. (Platygastroidea, Platygastridae); modified from MB 103503. Numbers indicate character numbers:characters states.

**Figure 7.**
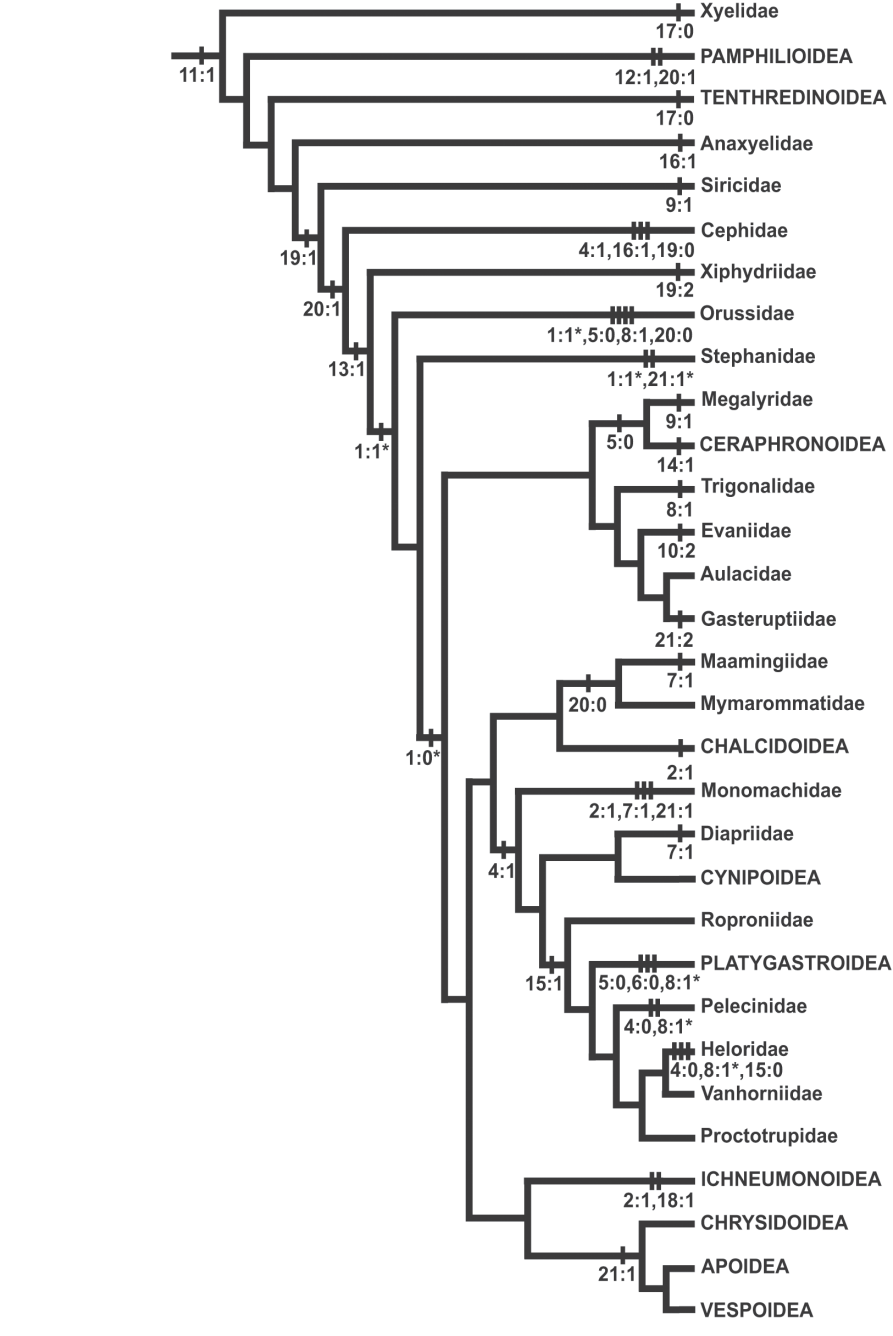
Selected character state changes mapped onto a phylogeny of Hymenoptera. Tree modified from Vilhelmsen et al. 2010a, fig. 69. Outgroup taxa have been removed and terminals have been collapsed to the family or superfamily level; Aulacidae, Diapriidae, Chrysidoidea and Vespoidea are treated as monophyletic. Not all character state changes shown; changes marked with a * have equally parsimonious alternative optimisations.

Heads of the taxa examined were detached from the rest of the body and the antennae and mouthparts were removed. The heads were examined in a dissection microscope and bisected in a parasagittal plane to allow the observation of internal features. In addition, SEM images of anterior and posterior views of the head of a number of taxa (see below) were downloaded from MorphBank (http://www.morphbank.net/) for a number of taxa and used to score as well as illustrate characters (see Figs 1-6). The data set was assembled in Mesquite (Maddison and Maddison 2007) and traced in the same program on a phylogeny of Hymenoptera produced by Vilhelmsen et al. (2010, fig. 69). A slightly modified version of this tree with the terminals collapsed at the family or superfamily level is used to illustrate the evolution of selected characters (Fig. 7).

## Material examined

Taxa for which Morphbank SEM images were available are indicated with ‘MB’ in the list below, followed by the image numbers.

### Outgroup

Neuroptera, Chrysopidae: *Chrysopa perla* (Linnaeus, 1758). Mecoptera, Panorpidae: *Panorpa communis* (Linnaeus, 1758). Lepidoptera, Micropterigidae: *Micropterix calthella* (Linnaeus, 1761).

### Hymenoptera

Xyeloidea, Xyelidae: *Macroxyela ferruginea* (Say, 1824) MB 102882, 102884, 102885.

Tenthredinoidea, Tenthredinidae: *Athalia rosae* (Linnaeus, 1758) MB 102486-88; *Notofenusa surosa* (Konow, 1905). Diprionidae: *Monoctenus juniperi* (Linnaeus, 1758). Pergidae: *Heteroperreyia hubrichi* Malaise, 1955.

Pamphilioidea, Pamphiliidae: *Onycholyda amplecta* (Fabricius, 1804) MB 102834, 102837.

Cephoidea, Cephidae: *Cephus pygmeus* (Linnaeus, 1767) MB 102672, 102946.

Siricoidea, Anaxyelidae: *Syntexis libocedrii* Rohwer, 1915. Siricidae: *Tremex columba* (Linnaeus, 1763) MB 102749, 134704; *Urocerus gigas* (Linnaeus, 1758).

Xiphydrioidea, Xiphydriidae: *Xiphydria camelus* (Linnaeus, 1758); *Xiphydria prolongata* (Geoffroy, 1758) MB 102708-9, 134719.

Orussoidea, Orussidae: *Orussobaius minutus* Benson, 1938; *Orussus abietinus* (Scopoli, 1763) MB 134672, 134675.

Ceraphronoidea, Ceraphronidae: *Ceraphron* sp. MB 78643, 78654. Megaspilidae: *Lagynodes* sp. MB 79001, 79018; *Megaspilus fuscipennis* (Ashmead, 1888); *Megaspilus* sp. MB 134646, 134648.

Chalcidoidea, Aphelinidae: *Cales noacki* Howard, 1907 MB 101253, 101256, 101328, 101330; *Coccophagus rusti* Compere, 1928 MB 101454, 101458. Chalcididae: *Acanthochalcis nigricans* Cameron, 1884; *Acanthochalcis* sp. MB 134808, 134812. Eulophidae: *Cirrospilus coachellae* Gates, 2000 MB 101190, 101192. Eurytomidae: *Eurytoma gigantea* Walsh, 1870 MB 101509, 101511. Mymaridae: *Gonatocerus ashmeadi* (Girault, 1915) MB 101615, 101617; *Gonatocerus morrilli* (Howard, 1908). Pteromalidae: *Cleonymus* sp. MB 101374-75, 101380-81; *Nasonia vitripennis* (Walker, 1836) MB 101734, 101736, 101738; *Spalangia nigripes* Curtis, 1839. Torymidae: *Megastigmus transvaalensis* (Hussey, 1956) MB 101672, 101672, 101709.

Cynipoidea, Cynipidae: *Diplolepis rosae* (Linnaeus, 1758) MB 71826-27; *Periclistus brandtii* (Ratzeburg, 1832) MB 72041-42. Figitidae: *Anacharis* sp.; *Melanips opacus* (Hartig, 1840) MB 75299; *Melanips* sp. MB 75329; *Parnips nigripes* (Barbotin, 1964) MB 80037, 80053. Ibaliidae: *Ibalia leucospoides* (Hochenwarth, 1785); *Ibalia rufipes* Cresson, 1879 MB 77314-15.

Evanioidea, Aulacidae: *Aulacus impolitus* Smith, 1991 MB 103222, 103243; *Pristaulacus strangaliae* Rohwer, 1917 MB 103739, 103747. Evaniidae: *Brachygaster minuta* (Olivier, 1792) MB 103249, 103257; *Evania albofascialis* Cameron, 1887 MB 134630, 134632; *Evaniella semaeoda* Bradley, 1908 MB 103427, 103443. Gasteruptiidae: *Gasteruption* spp. MB 103453; *Pseudofoenus* spp. MB 103788, 103810.

Ichneumonoidea, Braconidae: *Aleiodes terminalis* Cresson, 1869 MB 103161, 103186; *Doryctes erythromelas* (Brullé, 1846) MB 103274, 103330; *Orgilus gracilis* (Brues, 1908) MB 103649, 103655; *Rhysipolis* sp. MB 103818, 103843; *Urosigalphus* sp.; *Wroughtonia ligator* (Say, 1824) MB 103109, 103126, 134714, 134716. Ichneumonidae: *Dusona egregia* (Viereck, 1916) MB 103363, 103366, 134625, 134627; *Labena grallator* (Say, 1836) MB 103512, 103524; *Lymeon orbum* (Say, 1835) MB 103559, 103562; *Pimpla aequalis* (Provancher, 1880) MB 103694, 103718; *Zagryphus nasutus* (Cresson, 1868) MB 103134, 103149.

Megalyroidea, Megalyridae: *Dinapsis* spp.; *Megalyra fasciipennis* Westwood, 1832 MB 103568, 103589, 134657, 134660.

Mymarommatoidea, Mymarommatidae: *Mymaromma anomalum* (Blood & Kryger, 1922) MB 101797-98, 101800.

Platygastroidea, Platygastridae: *Archaeoteleia mellea* Masner, 1968 MB 101920, 101922, 103191, 103211; *Isostasius* sp. MB 103486, 103503; *Proplatygaster* sp. MB 103760, 103776, 134687, 134690; *Sparasion formosum* Kieffer, 1910 MB 103049, 103068; *Telenomus podisi* Ashmead, 1893 MB 103079, 103096.

Proctotrupoidea, Diapriidae: *Belyta* sp. MB 101879-80, 101882; *Pantolytomyia ferruginea* (Dodd, 1915) MB 79029; *Poecilopsilus* sp. MB 79100. Heloridae: *Helorus anomalipes* (Panzer, 1798); *Helorus* sp. MB 78665, 104116, 134637, 134639. Maamingidae: *Maaminga rangi* Early et al., 2001. Monomachidae: *Monomachus antipodalis* Westwood, 1874 MB 80081-82. Pelecinidae: *Pelecinus polyturator* (Drury, 1773) MB 79061, 134682-85. Proctotrupidae: *Austroserphus albofasciatus* Dodd, 1933 MB 78510, 103974, 103978; *Phaenoserphus* sp. MB 80116, 80121, 80147; *Proctotrupes* sp. Roproniidae: *Ropronia garmani* (Ashmead, 1898) MB 78872, 134697, 134700. Vanhorniidae: *Vanhornia eucnemidarum* Crawford, 1909 MB 78898, 134709, 134711.

Stephanoidea, Stephanidae: *Megischus* spp. MB 103607, 103620, 134650, 134653; *Schlettererius cinctipes* (Cresson, 1880).

Trigonaloidea, Trigonalidae: *Orthogonalys pulchella* (Cresson, 1867) MB 103661, 103682, 134678, 134680; *Taeniogonalos gundlachii* (Cresson, 1865) MB 103032, 103043.

Apoidea, Ampulicidae: *Ampulex compressa* (Fabricius, 1781) MB 134606, 134608; Crabronidae: *Pison chilense* Spinola, 1851 MB 102586-88; Sphecidae: *Stangeella cyaniventris* (Guérin-Méneville, 1831).

Chrysidoidea, Bethylidae: *Cephalonomia stephanoderis* Betrem, 1961 MB 102530-31, 102534. Chrysididae: *Chrysis angolensis* Radoszkowski, 1881. Plumaridae: *Plumarius* sp. Scolebythidae: *Ycaploca evansi* Nagy, 1975.

Vespoidea, Pompilidae: *Aporus niger* (Cresson, 1867). Rhopalosomatidae: *Rhopalosoma nearcticum* Brues, 1943. Sapygidae: *Sapyga pumila* Cresson, 1880 MB 102635, 102637. Vespidae: *Metapolybia cingulata* (Fabricius, 1804).

## Results and discussion: annotated character list

***1. Ocellar corona***

(0) absent (e.g., Figs 1A-C, 2B-D)

(1) present (Figs 1D, 2A)

The presence of a circlet or semicirclet of cuticular teeth around the median ocellus is a ground plan feature of both Orussidae (Vilhelmsen 2003) and Stephanidae (Aguiar 2001). Given the phylogenetic position in Vilhelmsen et al. (2010a) of these two families as successive outgroups to the remainder of the Apocrita and all Apocrita, respectively, it is equally parsimonious to assume that the ocellar corona has evolved independently in Orussidae and Stephanidae or in the common ancestor of Orussidae + Apocrita, subsequently to become lost in most of the latter (see also Vilhelmsen and Turrisi 2011).

***2. Supraantennal grooves or depressions***

(0) absent (e.g., Figs 3A-B, 4B, D)

(1) present (Figs 3C, 4A)

Grooves or depressions, also called scrobes, are prominent above the antennal foramina in many Chalcidoidea and apparently belong to the ground plan of the superfamily. However, they were not observed in *Gonatoceros* (Mymaridae). The Mymaridae have been suggested to be the sister group of the remainder of the Chalcidoidea (e.g., Gibson 1986, Gibson et al. 1999), a result that was recently corroborated by Heraty et al. (2011), but not by Davis et al. (2010) or Vilhelmsen et al. (2010a), were the Mymaridae were nested deeply within Chalcidoidea. The scrobes serve to accommodate the scapes of the antennae. Less well developed depressions are observed in a number of apocritan taxa, especially among the Ichneumonoidea, where they also seem to be a ground plan feature. They have been assigned the same character state as the more prominent grooves in Chalcidoidea.

***3. Notch on the inner margin of the eye***

(0) absent (e.g., Figs 3A, C-D)

(1) present (Fig. 3B)

A prominent notch is present on the inner margin of the eye in some Aculeata (especially Vespoidea) and Ichneumonoidea, but does not seem to be a ground plan feature of either of these taxa.

***4. Hairs on eyes***

(0) absent or indistinct, as most as long as diameter of ommatidium (e.g., Figs 3B, C)

(1) distinct setae longer than ommatidium present on at least part of eye (Figs 3A, D)

Among the non-apocritan lineages, distinct hairs on the eyes (between the ommatidia) were only observed in *Cephus* (Cephidae). In contrast, hairs are present in many apocritan taxa, especially among the Chalcidoidea and the Proctotrupomorpha s.str. (Vilhelmsen et al. 2010a). They probably evolved independently in these taxa as they are not present in *Maaminga* (Maamingidae) and *Mymaromma* (Mymarommatidae).

***5. Position of antennal foramina relative to eyes***

(0) below or level with ventral margin of eyes (Figs 2C, 3A, 4D)

(1) above ventral margin of eyes (Figs 1A, B, 2B, D, 3B, D, 4C)

***6. Position of antennal foramina relative to clypeus***

(0) equal or closer to clypeus than its own diameter (Figs 1A, B, 2B, C, 3A, B, 4D)

(1) further from clypeus than its own diameter (Figs 2D, 3D, 4C)

These two characters are partly overlapping, but it was decided score them separately to partition the information as finely as possible. Both characters are quite variable across the Hymenoptera, also within many superfamilies. Having the antennal foramina below the ventral margins of the eyes is apparently apomorphic for Orussidae and Platygastroidea, and possibly synapomorphic for Megalyridae + Ceraphronoidea.

***7. Frontal shelf***

(0) absent

(1) present, antennal foramina facing upwards (Early et al. 2001: fig. 6)

Early et al. (2001) proposed the presence of a prominent extension in lateral view of the frons to form a distinct shelf below the antennal foramina to be a putative synapomorphy of Diapriidae and Maamingidae; they noticed that a similar structure is also present in Monomachidae, Embolemidae (not included here) and some Ichneumonoidea. Furthermore, the shelf is absent from some Diapriidae (e.g., *Ismarus*). Vilhelmsen et al. (2010a) did not retrieve Diapriidae + Maamingidae in any of their analyses; however, Heraty et al. (2011) found support for a clade comprising Maamingidae, Monomachidae and at least part of the Diapriidae, Maamingidae and Monomachidae being sister groups within this clade.

***8. Inner margin of antennal foramen (ordered)***

(0) not distinctly raised compared to outer margin (Figs 1A, B, 2A, D, 3D)

(1) with distinct projection, raised compared to outer margin (Figs 2B, 4C)

(2) area between projections raised as well forming interantennal process (Figs 3A, 4B, D)

The presence of raised inner margins on the antennal foramina that might or might not be connected medially (states 1&2) are of scattered occurrence throughout the Hymenoptera. Having just the margins raised might be apomorphies of the Orussidae and Trigonalidae, as well as occurring in the groundplan of the Platygastroidea; within the latter, the projections have fused medially in *Archaeoteleia*, *Sparasion* and *Telenomus* (formerly placed in a separate family, the Scelionidae; see Sharkey 2007).

***9. Subantennal groove***

(0) absent or weakly developed (e.g., Figs 1A, B, 2A, D)

(1) present, distinct (Figs 1C, D, 2C)

The presence of a well developed groove extending from the antennal foramen and ventrally of the eye for accommodating the proximal part of the antenna is a putative apomorphy of Siricidae (Vilhelmsen 1997) and Megalyridae (Vilhelmsen et al. 2010b); it is also observed in many Orussidae (Vilhelmsen 2003) and Aulacidae (Turrisi and Vilhelmsen 2010), but it is not a ground plan feature of any of these families. Less developed subantennal grooves are observed in Stephanidae and Xiphydriidae (Vilhelmsen 1997), but these have been scored as state 0. There seems to be a strong correlation between the presence of well developed subantennal grooves and the adult having to emerge from a pupal chamber within wood (Vilhelmsen and Turrisi 2011).

***10. Epistomal sulcus (unordered)***

(0) absent or reduced, internal ridge absent (Figs 3D, 4C))

(1) present, distinct, usually with internal ridge (e.g., Figs 1A, 2B)

(2) present, not continuous medially, extends dorsally to antennal foramen

The epistomal sulcus and corresponding internal ridge separates the clypeus from the frons. Having these structures well developed and continuous medially is apparently a hymenopteran ground plan feature. The epistomal sulcus has been reduced a number of times within the Hymenoptera, notably in many Chalcidoidea and Cynipoidea, although it is uncertain whether the absence is a ground plan feature of either of these superfamilies. Having the sulcus well developed laterally, but discontinuous medially (state 2) is a putative apomorphy of Evaniidae.

***11. Clypeus***

(0) not inflected

(1) inflected, covering base of labrum (Figs 5A, C; Vilhelmsen 1996: figs 11-13)

In all Hymenoptera where this feature could be observed, the ventral margin of the clypeus is sclerotised also on its posterior, internal side, and the labrum is situated slightly posterior to it (Fig. 1A). Vilhelmsen (1996) stated this to be an autapomorphy for the Hymenoptera; this is corroborated here.

***12. Mandibular foramen***

(0) oral and mandibular foramen continuous (Fig. 5A)

(1) mandibular and oral foramina separated by subgenal sclerotisation (Fig. 5B)

The separation of the mandibular and oral foramina by sclerotisations continuous with the head capsule is a long established apomorphy of the Pamphilioidea (e.g., Königsmann 1977). A few of the apocritan taxa examined here also display this feature: *Ampulex* (Ampulicidae); *Mymaromma* (Mymarommatoidea).

***13. Occipital sulcus and ridge***

(0) absent (e.g., Figs 5C, 6B, D)

(1) present (Figs 5A, B)

The occipital sulci are present above the occipital foramen in the basalmost lineages of Hymenoptera and is definitely a ground plan feature of Hymenoptera (Vilhelmsen 1999). It is absent in Xiphydriidae, Orussidae and Apocrita, a putative synapomorphy for these taxa (Vilhelmsen 2001).

***14. Position, occipital foramen***

(0) approx. halfway between top of head and oral foramen (Figs 5A, B, 6A, B, D)

(1) distance from top of head to occipital foramen half or less than distance to oral foramen (Figs 5C, 6C)

The dorsally displaced occipital foramen is apparently an apomorphy of the Ceraphronoidea (Fig. 5C). It is also observed in a few other apocritan taxa (e.g., some Chalcidoidea).

***15. Position, posterior tentorial pits***

(0) adjacent/at level with occipital foramen/condyles (Figs 5B, 6A, B)

(1) considerably ventral to occipital foramen (Figs 5C, 6D)

The posterior tentorial pits are considered level with the occipital foramen when at least their dorsal ends reach the level of the ventral margin of the foramen (e.g., Fig. 5B). The ventrally displaced posterior tentorial pits are of scattered occurrence across the Apocrita. The trait is prominent in some Ceraphronoidea (Fig. 5C), where it is perhaps correlated with the dorsal position of the occipital foramen (see previous character). The ventrally placed tentorial pits also occur in the Pelecinidae, Platygastridae, Proctotrupidae, Ropronidae and Vanhornidae and might be interpreted as a ground plan feature/synapomorphy of a larger clade within the Proctotrupomorpha (see Fig. 7), but then the condition has to have reversed within the Heloridae and *Archaeoteleia*, *Sparasion* and *Telenomus* (Platygastroidea).

***16. Postoccipital bridge***

(0) absent

(1) present (Vilhelmsen 1999: figs 2D, 5C)

In contrast to the ventral sclerotisation (see character 17), this is an internal structure, formed by a sclerotisation between the insertion points of the ventral profurco-postoccipital muscles ventrally of the tentorial bridge (see Vilhelmsen 1999). Among the taxa examined here, it has only been observed in *Cephus* (Cephidae) and *Syntexis* (Anaxyelidae) and has probably evolved convergently in these two taxa.

***17. Sclerotisation between occipital and oral foramina***

(0) absent, foramina confluent (Fig. 5A)

(1) present, foramina separate (Figs 5B-D)

The various configurations of the ventral head sclerotisations in basal Hymenoptera were discussed at length in Vilhelmsen (1999). The external ventral head sclerotisation might take the form of either a hypostomal bridge, which is inferred to have formed by sclerotisation in situ of the area between the posterior tentorial pits (see Fig. 5B: 15:0) or a postgenal bridge (e.g., Fig. 6C), formed by the median extension of the genae into the hypostomal area, replacing the hypostomal bridge (Snodgrass 1960; Rasnitsyn 1969, 1988). These hypothetical transformation series seems to be based not so much on ontogenetic data as on observations of differences in the detailed configuration of various anatomical landmarks in the area between the occipital and oral foramina. The postgenal bridge is thus characterised by having the posterior tentorial pits separated from the oral foramen by a considerable distance and sometimes also by the presence of a median longitudinal, often hairy line (see Fig. 6C: 19:1) interpreted as the fusion line between the postgenae. In contrast, the hypostomal bridge does not display a narrow fusion line, although a broader hairy area might be present (Xiphydriidae; see Vilhelmsen 1999: figs 3C, D), and the posterior tentorial pits extend ventrally to close to the oral foramen. Given the variability of these landmarks across the Hymenoptera (see below) and the hypothetical nature of the transitions between them, it was decided not to differentiate between the two types of ventral sclerotisations here, but score just absence/presence of a sclerotisation. The variation in the configuration of lines on the ventral sclerotisation is coded in character 19. In this way, it is attempted to deal with the putatively phylogenetically relevant information from this region in a more objective way.

As it is scored here, the presence of a ventral head sclerotisation is optimized as a ground plan feature of the Hymenoptera, contrary to the inference of Vilhelmsen (1999); the absence of the sclerotisation in Xyeloidea and Tenthredinoidea is inferred to be secondary. However, this interpretation relies heavily on the condition of the selected outgroup taxa, most of which have a sclerotisation.

***18. Ventral sclerotisation configuration***

(0) at most oblique ventral to tentorial pits, not extending anteriorly (Figs 5D, 6A)

(1) horizontal ventral to pits and extending anteriorly (Fig. 6B)

This character has been scored inapplicable when the ventral sclerotisation (see previous character) is absent. The sclerotisation is inflected just below the tentorial pits in some apocritan taxa, forming the dorsal and anterior parts of a trough that accommodates the labiomaxillary complex. This condition was already noticed by Ross (1937) to be present in many Ichneumonoidea and is apparently an apomorphy of the superfamily, but it is also observed in Heloridae, Pelecinidae, Proctotrupidae, and Ropronidae.

***19. Longitudinal sulci on ventral head sclerotization (ordered)***

(0) none

(1) one median sulcus or hair line present, at least ventrally (Figs 5D, 6C, D)

(2) two sublateral sulci present, not merging ventrally (Figs 5B, 6C)

This character has been scored inapplicable when the ventral sclerotisation (see character 17) is absent. When a narrow median hair line is present, it has been scored state 1. In some cases (e.g., *Megastigmus* (Torymidae) Fig. 6C) both a median hair line and two sublateral sulci are present; these instances were scored as polymorphic (1&2). These longitudinal sulci and hair lines are some of the anatomical landmarks used to differentiate between the different types of ventral sclerotisations (see character 17), state 1 being indicative of a postgenal bridge, state 2 of a hypostomal bridge. This character is highly variable throughout the Hymenoptera. If treated as ordered, there is an unambiguous change from state 2 to state 1 in the common ancestor of Siricidae, Cephidae (which has state 0), Xiphydriidae (which has state 2 derived secondarily), Orussidae, and Apocrita (in which this character is very variable and displays few unambiguous changes). This corroborates the suggestion of Rasnitsyn (1988) that the postgenal bridge replaces the hypostomal bridge among basal Hymenoptera, albeit only once (with secondary reversals), not twice as he proposed (in the Siricidae and Vespina = Orussidae + Apocrita, respectively).

***20. Occipital carina***

(0) absent (Fig. 5A)

(1) present (Figs 5B-D, 6A-D)

The occipital carina extends dorsally and laterally of the occipital foramen, usually reaching the ventral margin of the head capsule close to the mandibular bases (but see following character). The presence of the occipital carina is not a ground plan character of the Hymenoptera but apparently evolved at least twice among the basal Hymenoptera, in the Pamphilioidea and (with reversals, e.g., in Maamingidae + Mymarommatidae) in the common ancestor of Cephidae, Xiphydriidae, Orussidae (where it is not a ground plan character, at least for the extant members of the family, see Vilhelmsen 2003) and Apocrita.

***21. Occipital carina configuration (unordered)***

(0) reaching ventral margin of head capsule (Figs 5B, 6A, B)

(1) not reaching ventral margin and not continuous medially (Figs 5C, 6C, D)

(2) continuous ventrally of occipital foramen (Fig. 5D)

This character has been scored inapplicable when the occipital carina is absent. In most Hymenoptera that have an occipital carina it reaches the ventral margin of the head capsule. Some taxa have the ventral ends of the occipital carina terminating before this, notably the Aculeata and Stephanidae. In a few apocritans, the ventral ends of the occipital carina joins medially below the occipital foramen, forming a continuous rim around the latter; this condition is a putative apomorphy of the Gasteruptiidae (Fig. 5D).

## Conclusion

The characters explored in the present paper show considerable variation across the Hymenoptera. For many of the characters, there is considerable variation within as well as between the families and superfamilies. The most unequivocal character support is usually displayed toward the distal ends of the tree as autapomorphies of families or superfamilies (the presence of a medially interrupted epistomal sulcus (char. 10:2) in Evaniidae; the presence of a dorsally displayed occipital foramen (char. 14:1) in Ceraphronoidea; the impression of the ventral sclerotisation below the occipital foramen to form a cavity for the labiomaxillary complex (char. 18:1) in Ichneumonoidea; having the occipital carina continuous ventrally of the occipital foramen (char. 21:2) in Gasteruptiidae); or autapomorphies of the Hymenoptera (the inflection of the clypeus; char. 11:1). Characters that corroborate more inclusive clades above the superfamily level are much rarer. An example of one such character is the absence of the occipital sulcus and ridge (char. 13:1) which has been lost in Xiphydriidae, Orussidae and Apocrita.

This pattern was even more obvious in the analyses of the much more comprehensive mesosomal data set assembled by Vilhelmsen et al. (2010a, fig. 67), where most relationships between the superfamily and ordinal levels were poorly, if at all, supported. This again emphasises the need to build comprehensive data sets for combined analyses to make progress with complicated phylogenetic problems like the higher level relationships of the Hymenoptera. The process of developing hymenopteran phylogenetics that Alexandr P. Rasnitsyn has done so much to further is continuing today, providing ever expanding insights into the evolutionary history of this megadiverse group.
